# Growth improvement of *Lolium multiflorum* Lam. induced by seed inoculation with fungus suspension of *Xerocomus badius* and *Serendipita indica*

**DOI:** 10.1186/s13568-019-0865-7

**Published:** 2019-09-12

**Authors:** Binghua Liu, Xinghong Liu, Fangchun Liu, Hailin Ma, Bingyao Ma, Wenxin Zhang, Lin Peng

**Affiliations:** 1 Shandong Academy of Forestry, 42, East Wenhua Road, Jinan, 250014 Shandong China; 2 Economic Forest Products Quality Inspection Test Center of State Forestry Administration (Jinan), Jinan, 250014 Shandong China; 3 Shandong Engineering Research Center for Ecological Restoration of Forest Vegetation, Jinan, 250014 Shandong China

**Keywords:** Growth promotion, *Lolium multiflorum* Lam., Root colonization, Seed germination, *Serendipita indica*, *Xerocomus badius*

## Abstract

In this study, a pot experiment was carried out in greenhouse to investigate the potentials of *Xerocomus badius* and *Serendipita indica* to penetrate and colonize roots of ryegrass (*Lolium multiflorum* Lam.) and to induce beneficial effects on seed germination and seedling growth. The results showed that *X. badius* and *S. indica* successfully colonized in the root system of *L. multiflorum* seedlings and the root colonization rate was 72.65% and 88.42%, respectively. By microscopy, the hyphae, chlamydospores and spores produced by *S. indica* were observed in roots cortex of *L. multiflorum* seedlings. In comparison with the non-inoculated seedlings, seedlings inoculated with *X. badius* and *S. indica* showed significant increase in growth parameters with plant height, basal diameter, biomass accumulation, relative growth rate, leaf relative water content and chlorophyll content. Also, we found that seedlings inoculated with *S. indica* exhibited a greater growth-promotion as compared with *X. badius*-inoculated seedlings. No significant influence of the two fungus application has been observed with respect to seed germination. It suggested that well establishments of mutualistic symbiosis between *L. multiflorum* and *X. badius* or *S. indica* were not so essential to seed germination but contributed highly to the survival and growth of the seedlings.

## Introduction

Plant growth-promoting fungi (PGPF) are heterogeneous group of nonpathogenic fungi that live freely in the root surface or the interior of the root itself or the rhizosphere and mediate improvements in seed germination, seedling vigor, plant growth, flowering and productivity of a wide range of host plants (Hossain et al. 2017). Plant growth promotion derived from plant-PGPF interactions mainly attributes to the production of plant growth-promoting compounds such as phytohormones and secondary metabolites, the enhanced nutrient availability, the amelioration of abiotic and biotic stresses, and the antagonism to phytopathogens (Bonfante and Genre 2010; Hock 2012; Tomer et al. 2016; Varma et al. 2017; Vijayabharathi et al. 2016; Yan et al. 2019).

Mycorrhizal fungi, one of the commonly occurring microorganism in soil associated with more than 80% of land plants (Bonfante and Genre 2010), play an increasingly vital important role in plant growth promotion, plant stress tolerance induction and agricultural sustainability (Hock 2012; Hossain et al. 2017; Rai et al. 2014; Varma et al. 2017; Yan et al. 2019). Mycorrhizal fungi establish stable and closer mutualistic symbiosis that generate a huge hyphal network in the soil and have prominent beneficial effect in improving the associated-plants absorption of soil water and nutrients. In exchange, the host plant provides the mycorrhizal fungi with a place to live and with carbohydrates that are essential to the completion of the fungal life cycle (Javeria et al. 2017).

Over the past decades, mycorrhizal-based commercial inoculants are sold world-wide as biofertilizers in a variety of formulations in agriculture, horticulture and forestry (Owen et al. 2015; Pathak and Kumar 2016; Rai et al. 2014; Tomer et al. 2016; Vujanovic and Germida 2017). Mycorrhizal fungal inoculation was basically implemented through seed sowing and seedling planting, and both of them have shown a significant increase in seedling growth and production (Hossain et al. 2017; Vijayabharathi et al. 2016). Given that healthy plants depend on healthy seeds, it directs attention to seed-fungus relationship, a key regulatory mechanism in germinating seeds and seedlings driven by fungus (Vujanovic and Germida 2017). Seed bio-priming with mycorrhizal fungi has been proved to be beneficial to seed germination, seedling survival and timely seedling establishment (Prasad et al. 2016; Varma et al. 2017), although detrimental effects of various fungi colonization on these parameters have been observed (Owen et al. 2015). Nevertheless, application of beneficial PGPF in agricultural practices promises to be a fundamental tool to achieve sustainability in crop production and feed a constantly growing global population (Hock 2012).

*Xerocomus badius* (current name: *Imleria badi*a) (Species Fungorum 2019) is one of the most popular wild edible ectomycorrhizal fungal species in mixed woodland, particularly common under spruce, pine and other conifer trees and occasionally appears under oak, beech, chestnut and many other deciduous broadleaf trees (https://www.first-nature.com/fungi/imleria-badia.php). Our previous study (has not been published) revealed that *X. badius* had a wide pH (5.00–9.00) tolerance especially the high alkali tolerance, and might have good adaptation to the alkali environments. *Serendipita indica* (formerly known as *Piriformospora indica*) is a mycorrhiza like axenically cultivable plant growth-promoting root endophytic fungus obtained from the rhizosphere soils of the woody shrubs *Prosopis juliflora* (Swartz) DC. and *Zizyphus nummularia* (Burm. fil.) Wt. & Arn. in the sandy desert soils of Rajasthan, India (Oelmüller et al. 2009; Varma et al. 1999). It can easily colonize with roots of many plants and shows the same functions as the typical arbuscular mycorrhizal fungi did in plant growth promotion and improvement of biotic and abiotic stress tolerance (Gill et al. 2016; Khademian et al. 2019; Unnikumar et al. 2013; Varma et al. 2012, 2017).

*Lolium multiflorum* Lam. is a cosmopolitan and dominant annual grass species that invades crops, pastures and grasslands, and it can survive in the salt-alkali environment (salinity 0–20‰, pH = 7–9) of the Yellow River delta region, Shandong, China (Chen et al. 2017). Thus, *L. multiflorum* can be used as vegetative cover to improve the ecological restoration and landscape in the salt-alkali regions, and also can be used as a forage grass when other plants suffer from winter kill. Previous researches showed that ryegrass (*Lolium* spp.) support arbuscular mycorrhizal fungi and endophytes and establish well mutualistic symbiosis with them (Gundel et al. 2011; Ponce et al. 2009). In view of these findings, we propose that *L. multiflorum* is expected to establish mutualistic symbiosis with *X. badius* and *S. indica*, that could be beneficial to the improvement of seed germination and seedling establishment.

The general objective of this research was (1) to verify the mutualistic symbiosis between *L. multiflorum* and *X. badius* or *S. indica* driven by seeds inoculation, and (2) to evaluate the effect of seed priming with fungus suspension on root colonization, seed germination, morphological and physiological responses of *L. multiflorum*. Our results would have important implications for the use of these fungi as inoculants on agricultural crops.

## Materials and methods

### Plant material, fungus strain and inoculum preparation

Seeds of *L. multiflorum* used in this study were obtained from Xinrui Seed Industry Limited Company, Jiangsu, China. The seeds of *L. multiflorum* were soaked in sterile water overnight and surface-sterilized by washing with 80% ethanol for 30 s and with 2% sodium hypochlorite (w/v) for 15 min; and then, the seeds were rinsed eight to ten times with sterile distilled water to remove the adhered chemicals.

*Xerocomus badius* and *S. indica* used in this study were obtained from China Forestry Culture Collection Center (Preservation No. cfcc5946, Beijing, China) and China General Microbiological Culture Collection Center (Preservation No. CGMCC3.17686, Beijing, China), respectively. The strains were maintained on Potato Dextrose Agar (PDA, pH = 6.5) slants and subcultured every 2 months. The slants were incubated at 26 °C ± 2 °C for 7 days in dark in an incubator (SPX-300B-G, BOXUN Ltd, Shanghai, China) and then stored at 4 °C.

*Xerocomus badius* and *S. indica* used for the experiment were initially grown on sterile PDA Petri dishes (diameter: 9.00 cm, reusable, Shanghai, China) in an inverted position for 7 days at 26 °C ± 2 °C in dark. And then, inoculums of the two strains was grown on Potato Dextrose Broth (PDB, pH = 6.5) in glass conical flasks. Generally, 4–5 fully colonized circular agar discs (from the Petrie plate cultures) were inoculated into each Erlenmeyer flask (250 mL, reusable, Shanghai, China) containing 150 mL PDB. The flasks were incubated at 26 °C ± 2 °C with constant shaking at 150 rpm on a rotary shaker (THZ-C-1, GUOWANG Ltd, Jiangsu, China) in dark. After 15 days of incubation on PDB, the fungus suspension was centrifuged at 6000 rpm for 5 min at 4 °C; and then, the pellet was resuspended in sterilized distilled water and was smashed with an agitator (WBL2521H, Midea, Guangdong, China). The spore concentration was adjusted to 5.0 × 10^6^ spores mL^−1^. The resulted suspension was passed through a bacterial filter for sterilization (Millex-GV, 0.22 μm filter Unit, Millipore) and if not used immediately, it would be preserved for up to 1 month at < 4 °C before application.

### Experimental design and growth condition

The present experiment was a factorial experiment in a completely randomized design with one factor (mycorrhiza fungal inoculation). Fungi were applied at three levels including no-inoculation (control), single inoculation with *X. badius* and *S. indica*, respectively.

According to Khademian et al. (2019), the sterilized seeds were inoculated by the resulted spore suspensions of *X. badius* and *S. indica* for 6 h at 26 °C ± 2 °C with constant shaking at 150 rpm on a rotary shaker (THZ-C-1, GUOWANG Ltd, Jiangsu, China) in dark. The control seeds were treated by sterilized distilled water under the same condition. Afterwards, fifteen seeds of each treatment were sown into each cubic pot (7.0 cm × 7.0 cm × 7.0 cm, the pot volume was adequate for root growth from the preliminary test) filled with 0.5 kg sterilized soil (local topsoil:sand:grass peat = 3:2:2 (v:v:v), pH = 6.57). The respective residual suspension was poured in each treatment (10 mL pot^−1^). All the pots were placed in a random position on a shelf in the greenhouse without supplementary illumination with night and day temperatures at 18 to 25 °C and relative humidity at 65–80% at the plant nursery of Shandong Academy of Forestry, Jinan (36°40′N, 117°00′E), Shandong Province, China.

These experiments were began on 16 April and were terminated on 14 May 2019. During the period of our experiments, all seedlings were watered daily with sterilized water and supplied weekly with sterilized 50% Hoagland’s solution (pH = 6.5) (Hoagland and Arnon 1950). To avoid edge effects, all pots were rotated weekly.

### Measurements

#### Seed germination

One week after sowing, cumulative seed germination number in different treatments were recorded and the seed germination rate (GR) which was defined as one hundred times the number of germinated seeds divided by the total number of seeds was calculated.

#### Root colonization

After 4 weeks of growth, seedlings of six pots from each treatment were analyzed for root colonization, respectively. The seedlings were uprooted, washed in running tap water to get rid of the planting medium and the root systems were cut off. Following procedure described by Lorenc et al. (2018), roots of *X. badius*-inoculated seedlings were stored in a fixative solution of 2.5% glutaraldehyde until further processed. From each sample, 30 root segments with main root length of 5 cm were randomly selected and mycorrhizal tips were counted and assessed under a stereomicroscope (Nikon SMZ800, Japan).

Root colonization measurement of *S. indica*-inoculated seedlings was modified according to Yaghoubian et al. (2014) and Anith et al. (2011). Specifically, the collected roots of *S. indica*-inoculated seedlings were cut into segments with length of about 1 cm and boiled in 10% KOH (w/v) for 5 min and subsequently neutralized with 1% HCl (v/v) for 10 min. Roots segments were then stained in 5% ink-vinegar (Sheaffer, Item No. 94231, BOM No. 728-8564-BLK, MMIX Sheaffer Pen Corporation, a division of BIC USA Inc., Shelton, CT 06484) solution (v/v) for 20 min and washed in distilled water for 1 min. Finally, the stained root was mounted on glass slide with distilled water and covered with glass cover, and then used for observation of hyphae, hlamydospores and spores under a compound microscope (Nikon Eclipse 50i, Japan) equipped with a high resolution QImaging camera system (MicroPublisher 5.0 RTV, QImaging, Canada) and the presence of chlamydospores in the cortex cells was documented for each root segment. Root colonization rate (RCR) was calculated according to the following formula:

$$RCR\left( \% \right)\, = \,\left( {N_{C} /N_{O} } \right)\, \times \,100,$$

where N_C_ and N_O_ are the number of root segments colonized and the total number of root segments observed, respectively.

#### Growth

At the end of the experiment, the final plant height (PH) and basal diameter (BD) were recorded from six repeating groups (ten seedlings for each repeat group) of each treatment. After harvesting, plant materials were divided into leaf, stem, and root portions to determine values for above-ground biomass (AB), root biomass (RB), total biomass (TB), and the root/shoot ratio (RSR, root biomass divided by shoot biomass). Materials were oven-dried at 70 °C to a constant weight and the final total dry biomass was then recorded. Relative height growth rate (HGR), relative basal diameter growth rate (BGR) and relative growth rate (RGR) were calculated by the standard formulas:

$$HGR = (lnH_{F} - lnH_{I} )/t,$$

$$BGR = (lnB_{F} - lnB_{I} )/t,$$

$$RGR = (lnW_{F} - lnW_{I} )/t,$$

where *H*_*I*_, *B*_*I*_ and *W*_*I*_ are the initial height, basal diameter, and dry biomass, respectively; *H*_*F*_, *B*_*F*_ and *W*_*F*_ are the final height, basal diameter, and dry biomass, respectively; and *t* is the time interval.

#### Leaf relative water content

Leaf relative water content (RWC) was determined gravimetrically. Briefly, the youngest fully expanded leaves from seedlings of each treatment were collected and weighed immediately for their fresh weight (FW); And then they were placed in distilled water in a closed container for 24 h at 4 °C in dark to obtain the turgid weight (TW). Dry weight (DW) was determined for the same leaves after oven-drying for 48 h at 70 °C. RWC was calculated as:

$$RWC\;(\% ) = \left[ {\left( {FW - DW} \right)/(TW - DW)} \right] \times 100$$

#### Chlorophyll content

For measurement of the photosynthetic pigments, 0.1 g fresh leaf material for fully expanded young leaves was ground to powder in liquid nitrogen, then chlorophyll was extracted with 20 mL of 80% acetone for 12 h at 4 °C in dark. Absorbances at 647 nm and 664 nm were determined with a Shimadzu UV-/vis spectrophotometer (Model UV2401PC, Shimadzu, Riverwood Drive, Columbia, MD, USA) and used to calculate leaf chlorophyll content (C_chl_) according to Guerfel et al. (2009).

### Statistical analyses

The experiments were performed through a completely randomized design. All the measurements were conducted in sextuplicate. Figures were drawn using the SigmaPlot 10.0 for Windows version (SigmaPlot for Windows Version 10.0, Systat Software Inc., USA). Values in the figures are means of six replicates. Bars represent the standard deviation. Statistical analysis was carried out using the SPSS-13.0 for Windows statistical software package (Standard released version 13.0 for Windows, SPSS Inc., IL, USA). Analyses of one-way variance (ANOVA) were used to evaluate the effects of different fungal inoculation on seed germination, root colonization, seedling growth, biomass accumulation and allocation, chlorophyll and water content of *L. multiflorum* seedlings. Tukey’s HSD (honestly significant difference) post hoc test (*P* ≤ 0.05) was performed to test the existence of statistical differences for the same parameter between seedlings inoculated by different fungus.

## Results

### Seed germination

**Fig. 1:**
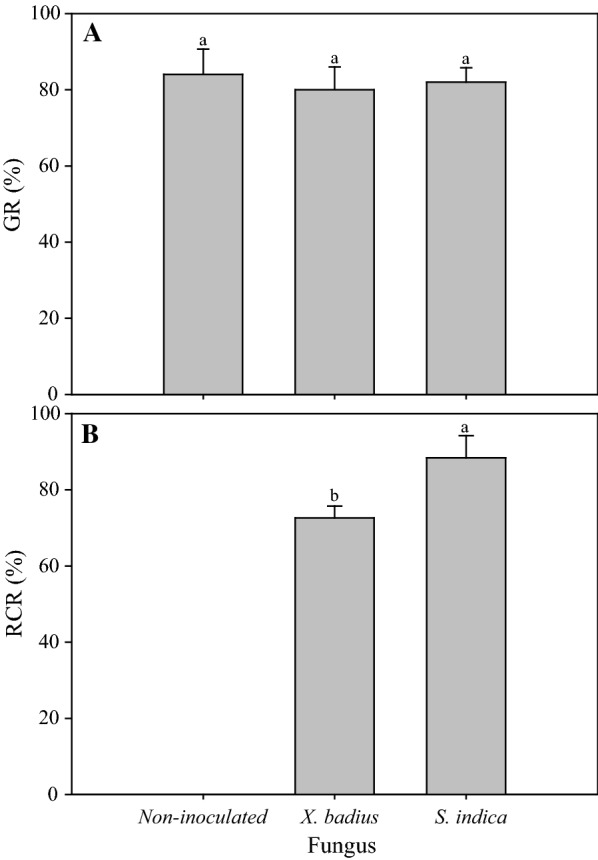
Effect of *X. badius* and *S. indica* inoculation on GR (**A**) and RCR (**B**) of *L. multiflorum* seedlings. *GR* seed germination rate, *RCR* root colonization rate. Values are means of six replicates. Bars represent the standard deviation. Lowercases show statistically significant differences for the same parameter among *L. multiflorum* seedlings inoculated by *X. badius* and *S. indic*a at *P* ≤ 0.05 based on Tukey’s HSD post hoc test

One-way variance analyses showed that fungal inoculation had no significant effect on seed germination of *L. multiflorum* (*P *> 0.05, Table 1). GR of *L. multiflorum* seeds inoculated by *X. badius* and *S. indica* was 80.00% and 82.33%, respectively; and it was not significantly differed from the non-inoculated seeds (84.00%) (*P *> 0.05, Fig. 1A).

**Table 1:** One-way ANOVA for effect of fungal inoculation on variables of *L. multiflorum* seedlings

Variables	*F*_FI_
AB	8.187***
BD	115.554***
BGR	78.947***
C_chl_	32.731***
GR	0.427
HGR	42.634***
PH	48.796***
RB	8.440*
RCR	20.448***
RGR	35.205***
RSR	6.981*
RWC	18.886**
TB	33.109***

AB, above-ground biomass; BD, basal diameter; BGR, relative basal diameter growth rate; C_chl_, leaf chlorophyll content; GR, germination rate; HGR, relative height growth rate; PH, plant height; RB, root biomass; RCR, root colonization rate; RGR, relative growth rate; RSR, the root/shoot ratio; RWC, leaf relative water content; TB, total biomass; *F*_FI_, effect of fungi inoculation

^*^, ^**^, ^***^, Significant at *P* ≤ 0.05, 0.01, and 0.001, respectively

### Root colonization

**Fig. 2:**
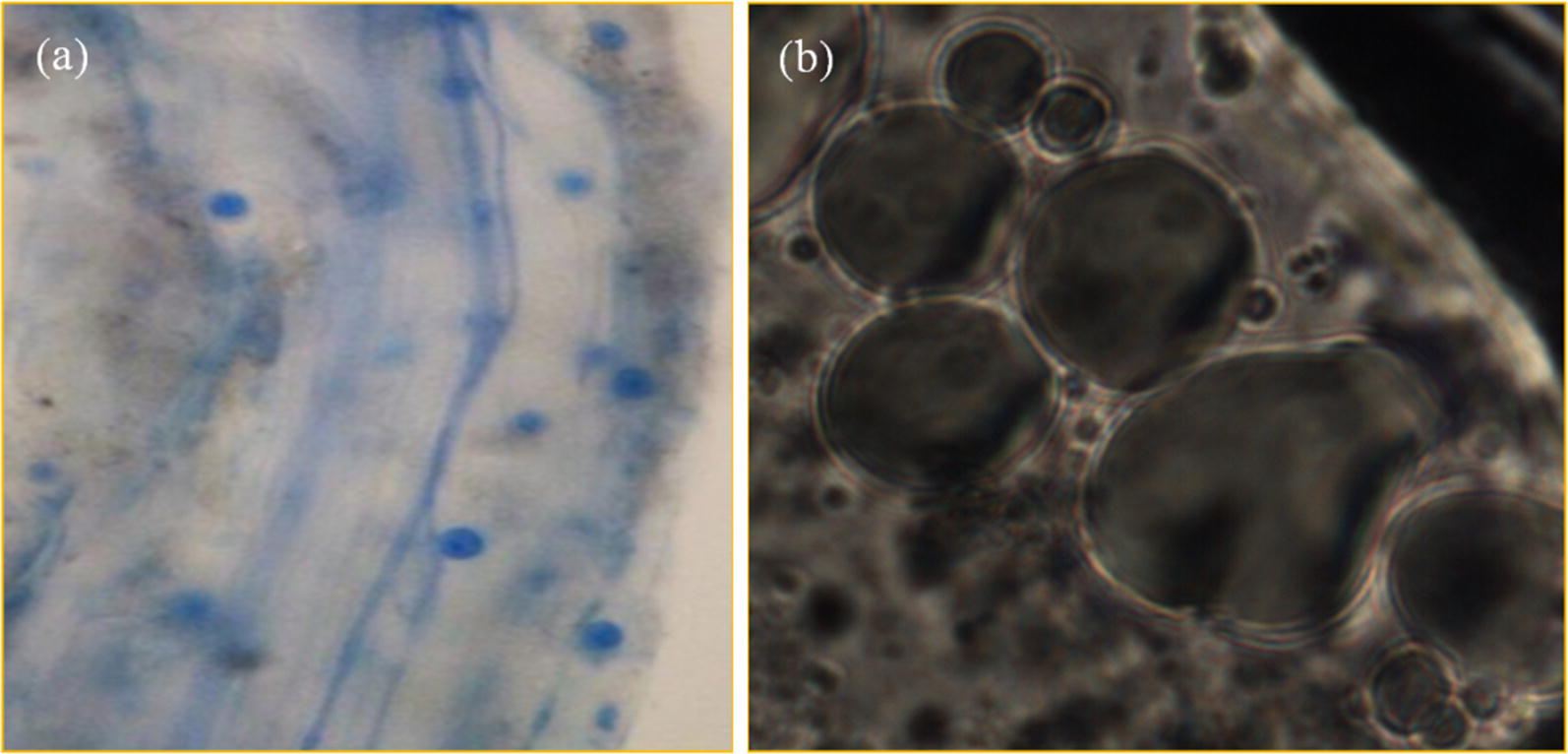
Hyphae, chlamydospores (**a**) and spores (**b**) produced by *S. indica* in roots cortex of *L. multiflorum* seedlings

Fungal inoculation had significant influence on RCR of *L. multiflorum* seedlings (*P* ≤ 0.05, Table 1). Four weeks after inoculation, significant differences in RCR were observed among *L. multiflorum* seedlings inoculated by different fungus (*P* ≤ 0.05, Fig. 1B). 72.65% inoculated seedlings of *L. multiflorum* were observed to be successfully infected by *X. badius* (Fig. 1B). Application of *S. indica* led to 88.42% colonization in roots of *L. multiflorum* seedlings (Fig. 1B). In microscopic analysis, the hyphae, chlamydospores (Fig. 2A) and spores (Fig. 2B) produced by *S. indica* were observed in roots cortex of *L. multiflorum* seedlings.

### Growth

**Fig. 3:**
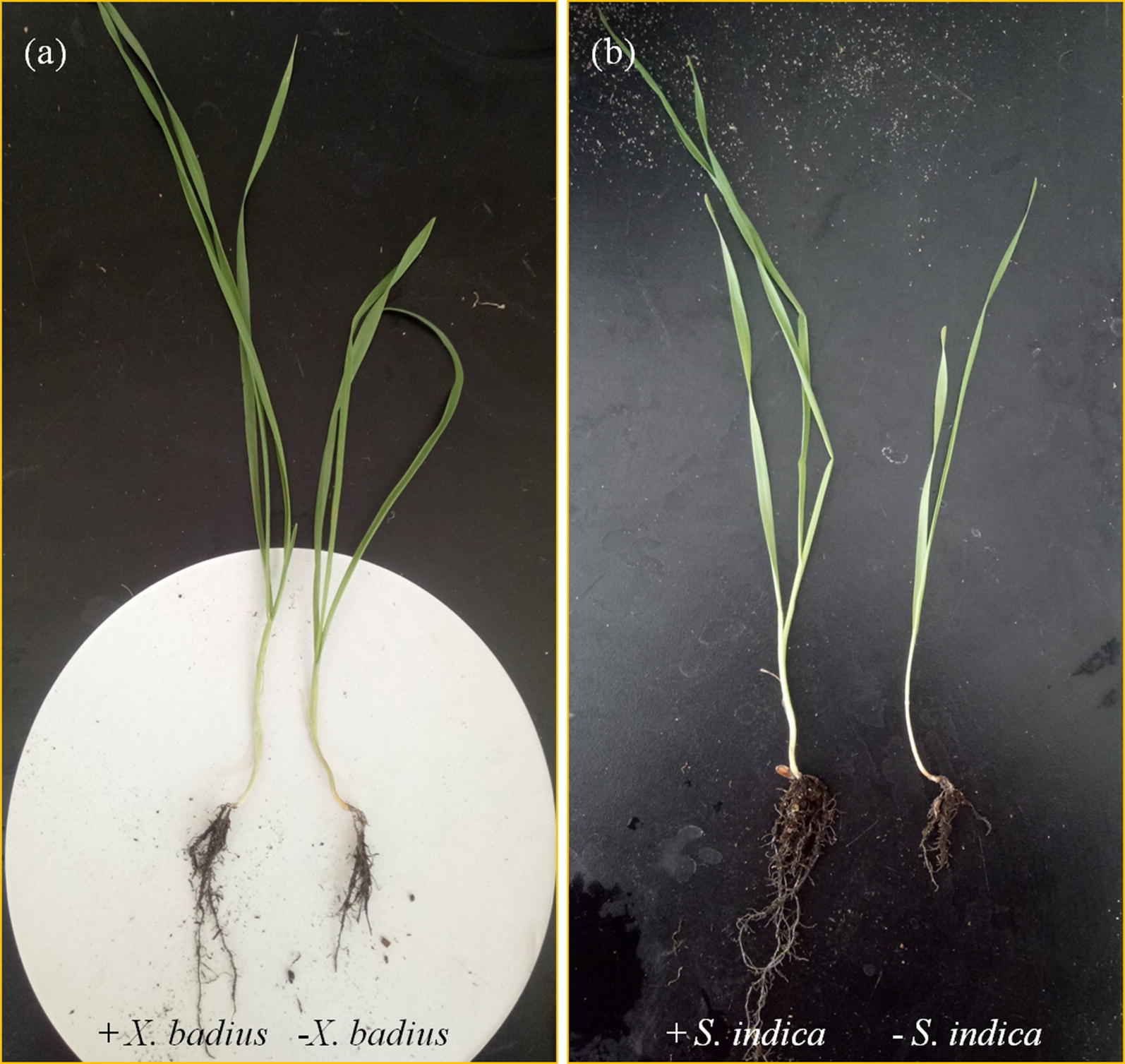
Typical phenotype of *L. multiflorum* seedlings 4 weeks after inoculation or non-inoculation with *X. badius* (**a**) and *S. indic*a (**b**)

According to the one-way variance analyses in Table 1, fungal inoculation had significant effect on PH (*P* ≤ 0.001), BD (*P* ≤ 0.001), AB (*P* ≤ 0.001), RB (*P* ≤ 0.05), TB (*P* ≤ 0.001) and RSR (*P* ≤ 0.05) of *L. multiflorum* seedlings. In comparison with the non-inoculated seedlings, overall growth including shoot and root of *L. multiflorum* was apparently improved by *X. badius* and *S. indica* as depicted in Fig. 3. With the fungal colonization, *L. multiflorum* seedlings were highly branched with increased number of tillers and numerous lateral rootlets; and robust increase in root hair development resulting in a bushy phenotype was observed in *X. badius*- and *S. indica*-inoculated seedlings (Fig. 3).

**Fig. 4:**
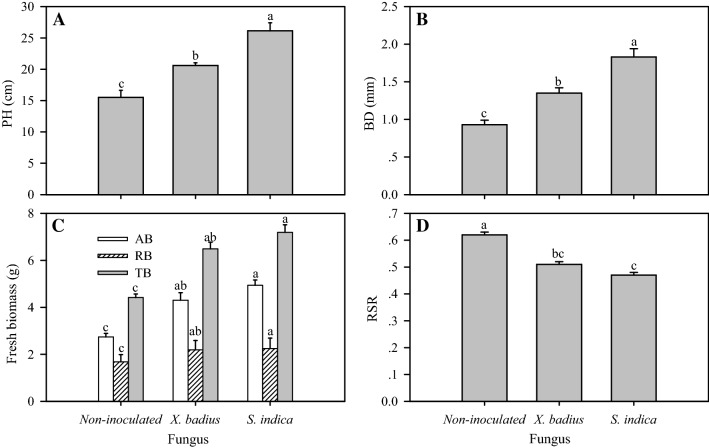
Effect of *X. badius* and *S. indica* inoculation on PH (**A**), BD (**B**), fresh biomass (**C**) and RSR (**D**) of *L. multiflorum* seedlings. *AB* above-ground biomass, *BD* basal diameter, *PH* plant height, *RB* root biomass, *RSR* root/shoot ratio, *TB* total biomass. Values are means of six replicates. Bars represent the standard deviation. Lowercases show statistically significant differences for the same parameter among *L. multiflorum* seedlings inoculated by *X. badius* and *S. indic*a at *P* ≤ 0.05 based on Tukey’s HSD post hoc test

Statistical analysis showed significant difference in PH and BD among *L. multiflorum* seedlings inoculated by different fungus (*P* ≤ 0.05, Fig. 4A, B). Seedlings inoculated by *S. indica* showed the highest value in PH (26.14 g) and BD (1.83 cm), followed by those inoculated by *X. badius* (PH and BD were 20.61 g and 1.35 cm, respectively) and non-inoculated seedlings (PH and BD were 15.52 g and 0.93 cm, respectively) (Fig. 4A, B). AB, RB and TB were significantly increased by fungal inoculation (*P* ≤ 0.05); and *S. indica* induced higher value in AB (4.94 g), RB (2.25 g) and TB (7.19 g) than *X. badius* (AB, RB and TB of the *X. badius*-inoculated seedlings were 4.30 g, 2.19 g and 6.49 g, respectively) (Fig. 4C). In addition, the extent of this response in AB was greater than that in RB, resulted in the significant (*P* ≤ 0.05) reduction in RSR which was 0.62, 0.51 and 0.47 for non-inoculated, *X. badius*-inoculated and *S. indica*-inoculated seedlings, respectively (Fig. 4D).

**Fig. 5:**
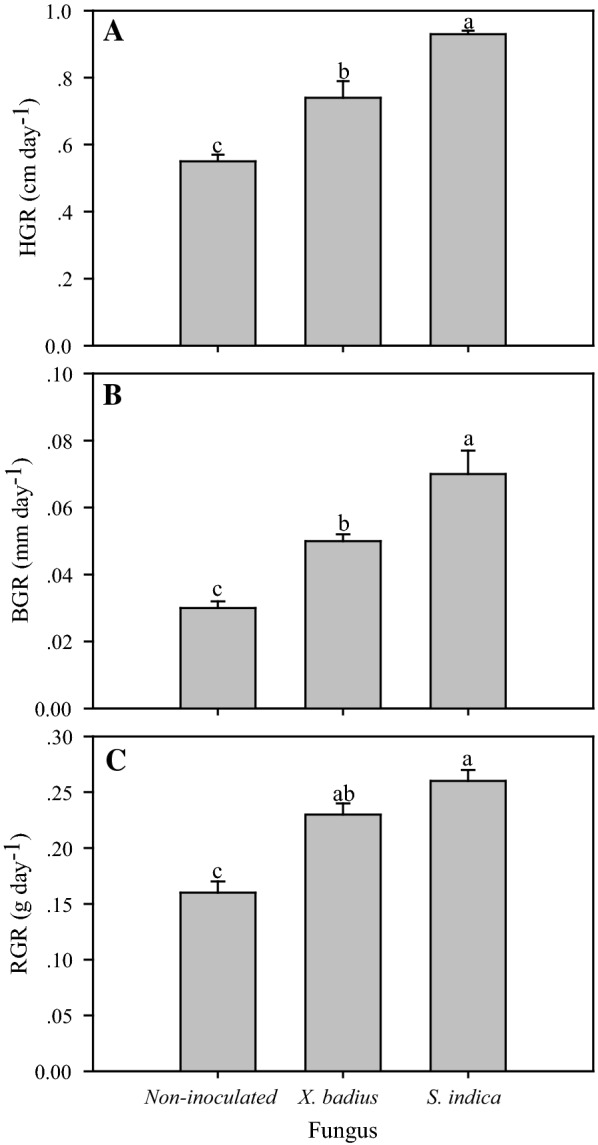
Effect of *X. badius* and *S. indica* inoculation on HGR (**A**), BGR (**B**), and RGR (**C**) of *L. multiflorum* seedlings. BGR: relative basal diameter growth rate, HGR: relative height growth rate, RGR: relative growth rate. Values are means of six replicates. Bars represent the standard deviation. Lowercases show statistically significant differences for the same parameter among *L. multiflorum* seedlings inoculated by *X. badius* and *S. indic*a at *P* ≤ 0.05 based on Tukey’s HSD post hoc test

One-way variance analyses showed that fungal inoculation had significant positive effects on HGR, BGR and RGR (*P* ≤ 0.001, Table 1) and significant differences in those variables were observed among *L. multiflorum* seedlings inoculated by different fungus (*P* ≤ 0.05, Fig. 5). *S. indica* induced the highest HGR (0.93 cm day^−1^), BGR (0.07 mm day^−1^) and RGR (0.26 g day^−1^), followed by *X. badius* (HGR, BGR and RGR were 0.74 cm day^−1^, 0.05 mm day^−1^, 0.23 g day^−1^, respectively) and non-inoculation (HGR, BGR and RGR were 0.55 cm day^−1^, 0.03 mm day^−1^, 0.16 g day^−1^, respectively) (Fig. 5).

### Leaf relative water content and chlorophyll content

**Fig. 6:**
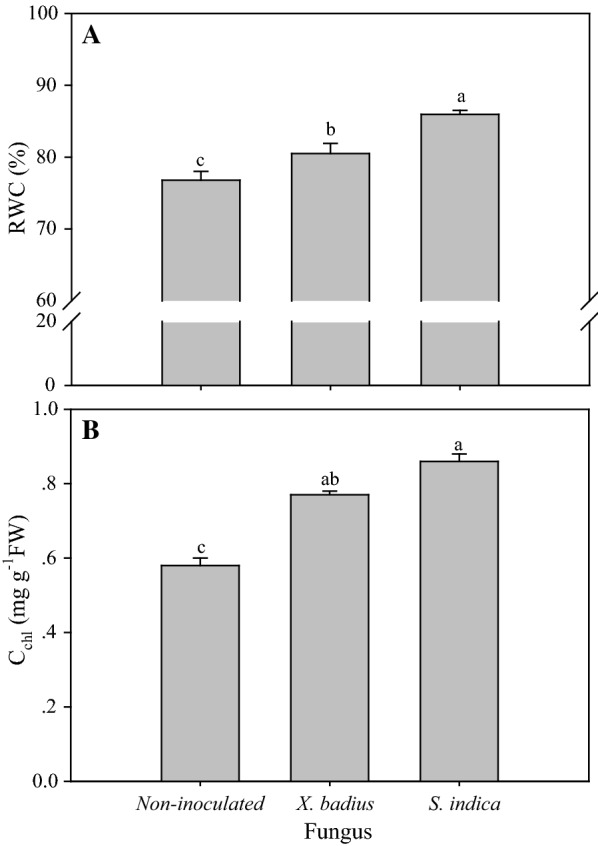
Effect of *X. badius* and *S. indica* inoculation on RWC (**A**) and C_chl_ (**B**) in leaves of *L. multiflorum* seedlings. C_chl_: content of leaf chlorophyll, RWC: leaf relative water content. Values are means of six replicates. Bars represent the standard deviation. Lowercases show statistically significant differences for the same parameter among *L. multiflorum* seedlings inoculated by *X. badius* and *S. indic*a at *P* ≤ 0.05 based on Tukey’s HSD post hoc test

Application of fungus had significant positive effect on leaf RWC (*P* ≤ 0.01) and C_Chl_ (*P* ≤ 0.001) (Table 1), and significant differences in RWC and C_Chl_ were observed among seedlings inoculated by different fungus (*P* ≤ 0.05, Fig. 6). *S. indica*-inoculated seedlings showed the highest RWC (85.95%) and C_Chl_ (0.86 mg g^−1^ FW), followed by *X. badius*-inoculated seedlings (RWC and C_Chl_ were 80.50% and 0.77 mg g^−1^ FW, respectively) and non-inoculated seedlings (RWC and C_Chl_ were 76.79% and 0.58 mg g^−1^ FW) (Fig. 6).

## Discussion

### Seed germination

Seed germination, a complex physiological process that starts with the uptake of water by the quiescent dry seed and terminates with radicle protrusion through the seed covering layers, is a critical step in the plant life cycle and is influenced by both abiotic and biotic factors (Rifna et al. 2019). Most crop seeds are routinely treated with a fungicidal seed dressing to improve seedling establishment and control early pathogenic infections, often accompanied by depress of seed germination, reduction of survival seedlings and detrimental effects on colonization by beneficial microorganisms (Pathak and Kumar 2016; Prasad et al. 2016). In recent years, fungal inoculation implemented through seed sowing has been widely confirmed beneficial to crop seed germination and seedling growth (Vujanovic and Germida 2017). For example, promotion effects on seed germination induced by PGPF inoculation with seeds have been reported on barley (Murphy et al. 2017), soybean (Waqas et al. 2012), rice (Doni et al. 2014), maize (Nayaka et al. 2010), orchid (Alghamdi 2019), and ect. During seed germination, the symbiotically associated fungi degrade cuticle cellulose resulting in alleviated retardation effect, provide available carbon and produce phytohormones (especially gibberellins and indole acetic acid) (Hock 2012; Hossain et al. 2017).

Not exactly so, our present study observed that inoculation by *X. badius* and *S. indica* with seeds had no significant positive effect on seed germination of *L. multiflorum* (*P *> 0.05, Table 1). Moreover, we found that the presence of fungi in seeds lead to earlier germination and greater survival of seedlings compared with the non-inoculated seeds. It suggested that the beneficial fungal associations were not so essential to seed germination but contribute highly to the survival and growth of the seedlings (that will be manifested by the following results). Similar results were also observed in bromeliad (Leroy et al. 2019), barley and oat (Murphy et al. 2017) inoculated by PGPF species.

### Root colonization

The degree of root colonization by fungi is a continuous plant trait and is influenced by external factors such as nutrient availability (Brundrett 2009; Soudzilovskaia et al. 2015). In our study, ectomycorrhizal fungus *X. badius* successfully colonized in the root system of *L. multiflorum* seedlings (the RCR was 72.65%) (Fig. 1B). In microscopic analysis, the mycorrhiza performed a typical characteristic of ectomycorrhizas with fungal hyphae surrounding the root tips and developing between epidermal cells but never enter the cell lumen. To our knowledge, this is the first report on the successful establishment of mutualistic symbiosis between *X. badius* and *L. multiflorum*, and the high fungal colonization might attribute to the great branching intensity of *L. multiflorum*. It is still a worthy project to research whether *X. badius* could induce regulatory mechanisms to resist stress conditions, in consideration of its physiological characteristics we observed in our previous research.

*Serendipita indica*, a symbiotic root endophyte, can colonize a broad spectrum of plant types including bryophytes, pteridophytes, gymnosperms and a large number of mono- and dicot plants (Franken 2012; Gill et al. 2016; Qiang et al. 2012; Unnikumar et al. 2013). The present study established a new symbiotic system between *L. multiflorum* and *S. indica* (RCR was 88.42%) (Fig. 1B). The hyphae, chlamydospores (Fig. 2A) and spores (Fig. 2B) produced by *S. indica* were observed in roots cortex of *L. multiflorum* seedlings. The colonization pattern of *S. indica* in *L. multiflorum* roots showed marked coincidence with the typical characteristics, actively colonizing into the root epidermis and cortex without any deeper expansion into the stele (Bonfante and Genre 2010).

### Growth

Plant growth is one of the most fundamental processes of vegetable kingdom, and plant growth rate has always been used as an important indicator of plant vitality (Dobbertin 2005). In the present study, application of fungi on seeds led to significant increase (*P* ≤ 0.05, Table 1) in HGR, BGR and RGR, that is consistent with earlier researches on *Helianthus annus* (Bagde et al. 2011) and *Brassica napus* (Su et al. 2017).

Increases in PH and BD of *L. multiflorum* seedlings were observed when suspension of *X. badius* and *S. indica* was applied to seeds in the present study (Fig. 4A, B). Moreover, a greater number of leaves/tillers with increased length and width were produced in the inoculated seedlings (results have not shown here), that could have increased the rate of photosynthesis and promoted the accumulation of carbohydrate and then resulted in the final rapid increase in AB and TB (Fig. 4C). This is in conformity with other studies where either fungus or fungal culture filtrate were applied to plants or seeds before sowing (Arunkumar and Shivaprakash 2017; Bagde et al. 2011; Khademian et al. 2019; Wu et al. 2019).

Above- and below-ground traits are coordinated along a whole-plant economics spectrum (de la Riva et al. 2016), root trait variation often shows a phylogenetic signal, particularly for mycorrhizal associations (Brundrett 2009). Besides the stimulation of shoot growth by the fungi, growth-promoting effect on roots was confirmed in the present study as manifested by the increase in RB (Fig. 4C). The fungal colonization resulted in an outstanding stimulation on root hair development with more lateral branching and resulted in a bushy root phenotype as depicted in Fig. 3. Researches on growth-promoting effect of *S. indica* manifested that the large increase in root hair was mainly due to the activation of auxin biosynthesis and signalling mediated by *S. indica* (Sirrenberg et al. 2007; Su et al. 2017). A well-developed bushy root system which boosted up the absorptive capacity of water and nutrition, played an important role in the beneficial effects of fungi on host plants (Arunkumar and Shivaprakash 2017; Bagde et al. 2011; Khademian et al. 2019; Wu et al. 2019; Su et al. 2017). Moreover, the growth-promoting effect of fungal colonization on AB was greater than that on RB, resulting in the significant (*P* ≤ 0.05) reduction in RSR (Fig. 4D). This might reflect an important regulatory mechanism, less increase in root absorption for much more biomass accumulation, for fungi to promote growth of the host plants (Wu et al. 2019).

In addition, significant difference in growth-promotion effect induced by the two different fungi was observed (*P* ≤ 0.05, Fig. 4). The promoted response induced by *S. indica* was more effective than that induced by *X. badius*.

### Leaf relative water content and chlorophyll content

Water is one of the necessary raw materials for photosynthesis that provides elections for the photosynthetic primary electron reaction by photolysis, and RWC is a useful indicator to expressing the balance between water supply to the leaf cells and transpiration rate (Mullan and Pietragalla 2012). Chlorophyll is the main component of photosynthetic pigments that are important to plants mainly for harvesting light and production of reducing powers, and C_chl_, an important indicator of photosynthetic capability, can directly affect photosynthetic potential and hence primary production (Gitelson et al. 2003). Symbiotic association between the tested fungi and *L. multiflorum* seedlings resulted in significant rise of leaf RWC (*P* ≤ 0.01) and C_chl_ (*P* ≤ 0.001) (Table 1), suggesting improvement of photosynthetic potential and hence great growth and biomass accumulation (as manifested above). *L. multiflorum* seedlings inoculated with *S. indica* showed higher RWC and C_Chl_ than *X. badius*-inoculated seedlings (*P* ≤ 0.05, Fig. 6). The promotion effect of *X. badius* on RWC might owe to the high surface area: mass ratio of ectomycorrhizas and their ability to penetrate microsites that are inaccessible for plant roots according to researches on other ectomycorrhizal fungi (Bonfante and Genre 2010). Positive effects of *S. indica* on RWC and C_Chl_ have been reported in many plants, especially under stressful conditions (Khademian et al. 2019; Khalid et al. 2018). Enhancement in leaf RWC induced by endophytic fungi of host plants mainly due to increasing of xylem pressure potential and fungal-produced exopolysaccharides that participate in higher water retention in the rhizosphere, leading to augmentation in water acquisition (Kohler et al. 2009).

In conclusion, both *X. badius* and *S. indica* successfully colonized in the root system of *L. multiflorum* seedling and induced beneficial effect on seedling growth as manifested by the significant increase in PH, BD, AB, RB, TB, HGR, BRG, RGR, RWC and C_chl_. Different from our original hypothesis, these establishments of mutualistic symbiosis were not so essential to seed germination according to the inconspicuous effect on GR. The hyphae, chlamydospores and spores produced by *S. indica* were observed in roots cortex of *L. multiflorum* seedlings, that showed marked coincidence with the typical characteristics.

To the best of our knowledge, this is the first report on the mutualistic symbiosis of *L. multiflorum* and *X. badius* or *S. indica* induced by seed priming with fungus suspension. Further field comparative experiments are needed to evaluate the effects of fungus inoculation at different stages of seedling development on a serious of responses with respect to water and nutrition absorption, vegetative and reproductive growth, maturation and yield, especially under stress conditions. These researches will provide useful references for application of fungi-based biofertilizers in agriculture, horticulture and even in forestry, and then the sustainable development of agriculture will be realized.

## Data Availability

The authors declare that all the data and materials used in this study are available.
